# Longitudinal Analysis of Contrasts in Gene Expression Data

**DOI:** 10.3390/genes14061134

**Published:** 2023-05-24

**Authors:** Georg Hahn, Tanya Novak, Jeremy C. Crawford, Adrienne G. Randolph, Christoph Lange

**Affiliations:** 1 Department of Biostatistics, Harvard T.H. Chan School of Public Health, Boston, MA 02115, USA; 2 Critical Care Medicine, Department of Anesthesiology, Boston Children’s Hospital, Boston, MA 02115, USA; 3 St. Jude Children’s Research Hospital, Memphis, TN 38105, USA

**Keywords:** contrasts, longitudinal data, gene expression, multiple organ dysfunction syndrome (MODS)

## Abstract

We are interested in detecting a departure from the baseline in a longitudinal analysis in the context of multiple organ dysfunction syndrome (MODS). In particular, we are given gene expression reads at two time points for a fixed number of genes and individuals. The individuals can be subdivided into two groups, denoted as groups *A* and *B*. Using the two time points, we compute a contrast of gene expression reads per individual and gene. The age of each individual is known and it is used to compute, for each gene separately, a linear regression of the gene expression contrasts on the individual’s age. Looking at the intercept of the linear regression to detect a departure from the baseline, we aim to reliably single out those genes for which there is a difference in the intercept among those individuals in group *A* and not in group *B*. In this work, we develop testing methodology for this setting based on two hypothesis tests—one under the null and one under an appropriately formulated alternative. We demonstrate the validity of our approach using a dataset created by bootstrapping from a real data application in the context of multiple organ dysfunction syndrome (MODS).

## 1. Introduction

In this article, we present novel methodology whose development was motivated by an application in the context of multiple organ dysfunction syndrome (MODS). The data that motivated this research are structured as follows. We are given gene expression reads at two time points for m∈N genes and n∈N individuals. The gene expression reads at the two time points are translated into contrasts of gene expression per individual and gene, thus effectively reducing the input to a scalar value per individual and gene. Moreover, we possess the age of each individual. The individuals can be subdivided into two groups, denoted as groups *A* and *B*. For instance, the two groups can be defined as those individuals who recovered from MODS (group *A*) and those who suffer from a condition called “prolonged MODS” (group *B*).

For each gene separately, we want to perform a linear regression of the gene expression contrasts on the individual’s age. As we are interested in a departure from the baseline, we look at the intercept of the linear regression. The aim of this work is to reliably single out those genes for which there is a difference in the intercept among those in group *A* and not in group *B*.

To approach this, we develop testing methodology based on two hypothesis tests. The two hypothesis tests are once under the null, and once under an appropriately formulated alternative. In an abstract setting, we are faced with two linear regressions—$L_{i}:y_{i}=\alpha_{i}a_{i}+\beta_{i}$, where $y_{i}\in R^{n_{i}}$, $a_{i}\in R^{n_{i}}$, and $\alpha_{i},\beta_{i}\in R$ for i∈{1,2}, where $n_{1}$ and $n_{2}$ are the sizes of the groups *A* and *B*, respectively. For group A, we wanted to test the hypothesis $H:\beta_{1}=0$ versus its complement $H^{\prime }:\beta_{1}\neq 0$. For group *B*, given some level λ, we test under the alternative, meaning $\tilde{H}:\beta_{2}>\lambda$ versus its complement ${\tilde{H}}^{\prime }:\beta_{2}\leq \lambda$.

The aforementioned tests are carried out on both groups for each gene contrast under consideration. Since there are m∈N genes, we have to use a multiple testing correction across the 2m *p*-values obtained by testing $H_{1}$ and $H_{2}$ for all *m* genes.

We demonstrate the validity of our approach using a simulated dataset in the context of multiple organ dysfunction syndrome (MODS) [1]. The simulated dataset has the purpose of showcasing the new methodology, and it is generated by bootstrapping from real MODS data of the Pediatric Intensive Care Influenza (PICFLU) investigators group [2,3]. As the purpose of the present article is to introduce the specific testing scenario and one possible solution approach, the usage of simulated data is valid. An application to real MODS data and the subsequent biological interpretation of the results are deferred to a separate publication.

The article is structured as follows. We start with a brief literature review in Section 1.1. Section 2 gives details on the dataset under the investigation and introduces the methodology we employ to solve our testing problem. This methodology can be subdivided into a series of (mathematical) components; for instance, the calibration of the alternative, the precise calculation of *p*-values, and the hypothesis tests being carried out. A demonstration of the methodology on simulated data can be found in Section 3. The article concludes with a discussion in Section 4.

Throughout the entire article, we denote with $Y_{\cdot ,j}$ the *j*’th column of a matrix *Y*.

### 1.1. Literature Review

While, initially, studies often compared gene expression data between distinct groups at fixed time points, there is a growing literature which considers time dependent expression data, meaning studies which extract insights from mRNA (or similar) samples collected at successive time points.

The identification of differentially expressed genes in a time course study is an active area of research. Starting with [4], the authors consider a two-way analysis of variance (ANOVA) approach combined with a permutation test to obtain *p*-values, where both group and time are the main factors. Their aim is to quantify the group effects (via permutations over all levels of the group labels for a fixed time point) and the time effects separately. The proposed approach to handling this problem is a two-stage model which first removes the time effect and then looks at the group effect. The given model is linear and the effects are determined via hypothesis testing with multiplicity correction.

In another work [5], the authors propose to identify differentially expressed genes in a time course study using a parametric model for the expression values in connection with a false discovery rate approach. In particular, the authors aim to detect changes in either a single biological group or differences in expression over time between two or more groups. The proposed method, called EDGE, is designed for inputs with or without replicates, and for both single group and multiple group tests. The trajectories of expressed genes are modelled with cubic splines and their goodness-of-fit to the model under both the null and alternative is evaluated, where the distributions are approximated via bootstrapping.

Another contribution in the literature [6] considers the questions of how to combine simultaneous inferences across multiple time points, as well as how to best control for multiplicity while accounting for the strong dependence between measurements. The authors formulate a decision-theoretic framework in which a gene is significant if a certain combination of null hypotheses is rejected at a given level. The focus of the authors is specifically on the optimal combination of testing at multiple time points, suitable multiplicity correction, and dependence among hypotheses. The hidden Markov model of [7] is generalized to capture the temporal correlation in the gene expression data.

In a similar fashion, in [8], the authors study the question of how to identify genes associated with a biological process in data having multiple time points, though not necessarily coming from the same individuals. They propose an approach based on functional principal component analysis (FPCA) in connection with hypothesis testing that allows one to incorporate high dimensionality, a low number of replicates, missing values, and measurement errors or time correlations. In their model, the parametric form of the null hypothesis is unknown, and thus has to be approximated via permutations.

In contrast to the methodology presented in our article, which aims to identify gene contrasts showing a departure from the baseline in one group and not another, the aforementioned publications differ from ours in that they either aim to identify different group and time effects, consider two null hypotheses, or are based on functional principal component analysis.

A second line of research available in the literature aims to process expression profiles with graphical methods as opposed to hypothesis testing. For instance, an algorithm to increase the temporal resolution of expression measurements and an application to skeletal muscle differentiation can be found in [9]. The algorithm is essentially a pipeline to process cell expression profiles, which includes dimensionality reduction via independent component analysis, the construction of a minimum spanning tree (MST), and the subsequent computation of the longest path through the MST, which corresponds to the longest sequence of transcriptionally similar cells.

In the exploratory study of [10], the authors combine libraries of single-cell RNA-Seq for primary mouse bone marrow-derived dendritic cells (DCs) and find substantial variation between identically-stimulated DCs.

Finally, there is literature on software tutorials for gene expression analysis with different time points, which, however, does not present new methodology. A workflow for the statistics computer software R, for the purpose of analyzing data from a micro-array time-course experiment, is presented in [11]. The tutorial considers quality control and normalization, the identification of genes that are differentially expressed, the clustering of genes into distinct temporal patterns, and the biological interpretation of the clusters. Some of the examples considered in the tutorial cover the exposure of mice to three different strains of influenza and lung tissue data collected at 14 time-points after infection.

## 2. Methods

This section introduces the methodology pipeline we developed for the problem of longitudinal analysis of contrasts, which was briefly outlined in Section 1. We present our approach as a series of steps. We start with a mathematical abstraction of the problem in Section 2.1. A high level summary of our approach is given in Section 2.2 before giving details on the calibration of the alternative hypothesis (Section 2.3), the calculation of *p*-values (Section 2.4), and the testing of all generated hypotheses (Section 2.5). We conclude with a note on how to report findings (Section 2.6).

### 2.1. Problem under Investigation

**Figure 1:**
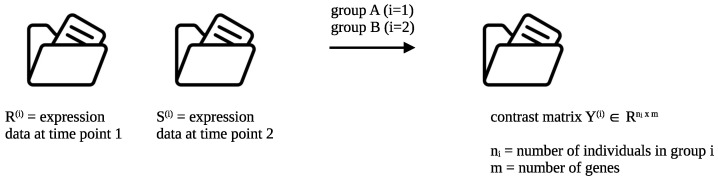
Preparation of the datasets $Y^{\left(i\right)}$ containing the contrast data for group *A* (i=1) and group *B* (i=2). The contrast data matrix $Y^{\left(i\right)}=R^{\left(i\right)}-S^{\left(i\right)}$ is obtained by computing the componentwise difference between the data at the two input timepoints $R^{\left(i\right)}$ and $S^{\left(i\right)}$.

Denote the sizes of the groups *A* and *B* as $n_{1}\in N$ and $n_{2}\in N$, respectively, and let $n=n_{1}+n_{2}$. The first step is to reduce the input to a single contrast matrix $Y^{\left(i\right)}\in R^{n_{i}\times m}$ for each group i∈{1,2}, where m∈N is the number of genes. This effectively reduces the two endpoint problem we consider to a single endpoint. The matrices $Y^{\left(i\right)}$ for group i∈{1,2} contain the contrast for individual *k* and gene *j* at position (k,j). This is visualized in Figure 1.

Formally, we are given two matrices $R^{\left(i\right)},S^{\left(i\right)}\in R^{n_{i}\times m}$ for i∈{1,2}, where the matrices $R^{\left(i\right)}$ and $S^{\left(i\right)}$ contain expression data per individual (row) and gene (column). Using this data, we compute a contrast matrix $Y^{\left(i\right)}=R^{\left(i\right)}-S^{\left(i\right)}\in R^{n_{i}\times m}$ per group, where i∈{1,2}. The ages of the individuals in the two groups shall be given as a vector $a_{i}\in R^{n_{i}}$ for i∈{1,2}.

For each gene j∈{1,…,m}, we perform two linear regressions of the contrast on the age with intercept, that is, we perform $L_{i,j}:Y_{\cdot ,j}^{\left(i\right)}=\alpha_{i,j}a_{i}+\beta_{i,j}$, where $\alpha_{i,j},\beta_{i,j}\in R$ for i∈{1,2}. For group *A* (meaning i=1), we want to test the hypothesis $H_{j}:\beta_{1,j}=0$ versus its complement $H_{j}^{\prime }:\beta_{1,j}\neq 0$ for each j∈{1,…,m}. For group *B* (meaning i=2), we test under the alternative given some level $\lambda_{j}$ for each gene *j*. To be precise, we test ${\tilde{H}}_{j}:\beta_{2,j}>\lambda_{j}$ versus its complement ${\tilde{H}}_{j}^{\prime }:\beta_{2,j}\leq \lambda_{j}$.

### 2.2. Summary of the Approach

We aim to identify those individuals that are in both group *A* and *not* in group *B*, and whose gene expression reads are explained by a linear model that includes the age without intercept (see Section 1). Since the selection criterion is different for the two groups, the formulation of two simple hypothesis tests is chosen as it provides a valid framework to draw statistical inference.

**Figure 2:**
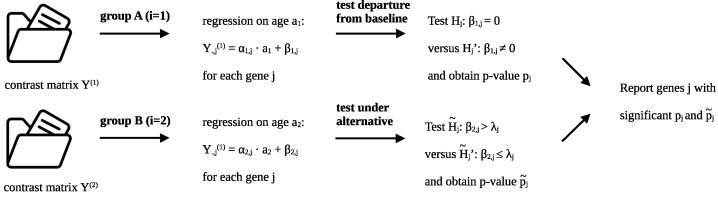
Summary of the testing pipeline. For each of the two groups *A* (i=1) and *B* (i=2), the same regression is carried out for each gene j∈{1,…,m}, using the data across the population of individuals ($n_{1}$ for group *A*, and $n_{2}$ for group *B*). However, the intercepts $\beta_{1}$ and $\beta_{2}$ are tested in two different ways. For group *A*, the departure from the baseline is tested, meaning $H_{j}:\beta_{1,j}=0$ for each gene *j*. For group *B*, the hypothesis ${\tilde{H}}_{j}:\beta_{2,j}>\lambda_{j}$ is tested under the alternative for an appropriately selected $\lambda_{j}$ and for all j∈{1,…,m}. The reported genes *j* must have significant *p*-values $p_{j}$ and ${\tilde{p}}_{j}$.

The complete approach we use to identify such genes is summarized in Figure 2. Specifically, we start with the two contrast matrices $Y^{\left(1\right)}$ and $Y^{\left(2\right)}$ for groups *A* and *B*, respectively. For both groups, the linear model of Section 2.1 is fitted separately to each gene j∈{1,…,m}. For group *A*, the one for which we want to test for an intercept of zero, we test the null hypothesis $H_{j}:\beta_{1,j}=0$ versus the complement $H_{j}^{\prime }:\beta_{1,j}\neq 0$ (see Section 2.1) for each gene j∈{1,…,m}. For group *B*, we test under the alternative ${\tilde{H}}_{j}:\beta_{2,j}>\lambda_{j}$ versus its complement ${\tilde{H}}_{j}^{\prime }:\beta_{2,j}\leq \lambda_{j}$ for each gene j∈{1,…,m}. Details on the calibration of the alternative are deferred to Section 2.3. In total, we thus observe 2m *p*-values (computed as described in Section 2.4), one per group and per gene j∈{1,…,m}. As a final step, we evaluate the resulting 2m*p*-values to single out only those genes j∈{1,…,m} for which both $H_{j}$ (having *p*-values $p_{j}$) and ${\tilde{H}}_{j}$ (having *p*-values ${\tilde{p}}_{j}$) are rejected. Details on this multiple testing problem are given in Section 2.5.

### 2.3. Calibration of the Alternative

To have a well-defined alternative ${\tilde{H}}_{j}$, it is necessary to specify a level $\lambda_{j}\in R$ for each gene j∈{1,…,m}. This means we consider an individual level for each test under the alternative. The calculation of the level under the alternative is performed as follows.

First, we compute the linear regression fit $L_{i,j}:Y_{\cdot ,j}^{\left(i\right)}=\alpha_{i,j}a_{i}+\beta_{i,j}$ for i=2 and all j∈{1,…,m}. We only consider the case i=2 here, since the calibration of the alternative applies to the hypotheses ${\tilde{H}}_{j}$ only. Second, for the fitted coefficients $\alpha_{2,j}$ and $\beta_{2,j}$, we compute the $R^{2}$ measure of the goodness-of-fit, and then scale the intercept $\beta_{2,j}$ (in either direction) until it explains only a proportion 0<π<1 of the original $R^{2}$. Computationally, since the $R^{2}$ measure decreases monotonically for misspecified coefficients, a binary search can be used across an interval of values for $\beta_{2,j}$ until the value explaining the fraction π of the initial $R^{2}$ is found. The value of $\beta_{2,j}$ explaining the proportion π of the initial $R^{2}$ is recorded as the level $\lambda_{j}$ for each j∈{1,…,m}. In our simulations in Section 3, we use π=0.35. This choice is arbitrary but motivated by practical applications [3].

### 2.4. p-Value Calculation

After having conducted all linear regressions of the null $H_{j}$ (see Section 2.1), we compute the *p*-values of the contrasts as described in [12,13]. Let $q_{d}\left(\delta \right)$ denote the lower δ quantile of the t-distribution with *d* degrees of freedom.

In particular, let $t_{i,j}$ be the t-value statistic calculated for each linear regression $L_{i,j}$. For $H_{j}$, that is, in the scenario of group *A* (meaning i=1), when the level is $\lambda_{j}=0$, the distribution of $t_{1,j}$ is a t-statistic with $n_{j}-2$ degrees of freedom. Therefore, the (two-sided) *p*-value of the contrast is given as $p_{j}=2q_{n_{j}-2}\left(t_{1,j}\right)$.

For the alternative ${\tilde{H}}_{j}$, that is, in the scenario of group *B* (meaning i=2), we test ${\tilde{H}}_{j}:\beta_{2,j}>\lambda_{j}$. This is equivalent to testing ${\tilde{H}}_{j}:\beta_{2,j}-\lambda_{j}>0$. Therefore, the (one-sided, upper tail) *p*-value of the contrast is given as ${\tilde{p}}_{j}=1-q_{n_{j}-2}\left(t_{2,j}-\lambda_{j}\right)$.

### 2.5. Multiple Hypothesis Testing

The *p*-value calculations of Section 2.4 result in 2m *p*-values, a vector $\left(p_{1},\ldots ,p_{m}\right)$ for $\left(H_{1},\ldots ,H_{m}\right)$, and a vector $\left({\tilde{p}}_{1},\ldots ,{\tilde{p}}_{m}\right)$ for $\left({\tilde{H}}_{1},\ldots ,{\tilde{H}}_{m}\right)$. We are interested in evaluating those *p*-values in such a way as to find those indices j∈{1,…,m} for which both $H_{j}$ and ${\tilde{H}}_{j}$ are rejected.

As we evaluate several hypotheses at the same time, a multiple testing correction is necessary. We consider two classical options in the remainder of the article, the Bonferroni correction [14] to control the Familywise Error Rate (FWER), and the Benjamini–Hochberg procedure to control the False Discovery Rate (FDR) [15]. Either procedure can be used, so long as one discloses that the reported significances are with respect to FWER or FDR control, respectively.

To stay conservative, we fix a testing threshold α∈(0,1) which is prespecified by the user. We evaluate the hypotheses of both groups *A* and *B* separately, using a testing threshold of α/2 for each to keep the overall type I error under control. We denote with $R_{H}^{B}$ and $R_{H}^{BH}$ the sets of rejected hypotheses among $H=(H_{1},\ldots ,H_{m})$ based on the *p*-values $\left(p_{1},\ldots ,p_{m}\right)$ for the Bonferroni correction and Benjamini–Hochberg procedure, respectively. Similarly, we denote with $R_{\tilde{H}}^{B}$ and $R_{\tilde{H}}^{BH}$ the sets of rejected hypotheses among $\tilde{H}=\left({\tilde{H}}_{1},\ldots ,{\tilde{H}}_{m}\right)$ based on the *p*-values $\left({\tilde{p}}_{1},\ldots ,{\tilde{p}}_{m}\right)$ for the Bonferroni correction and the Benjamini–Hochberg procedure, respectively.

### 2.6. Reporting the Findings

After evaluating all 2m hypotheses, we determine whether there exist one or more indices j∈{1,…,m} such that both $H_{j}$ and ${\tilde{H}}_{j}$ are rejected. In this case, we report these indices as findings. If no such index exists, we report an empty set.

To be precise, we report the hypotheses in the set $R_{H}^{B}\cap R_{\tilde{H}}^{B}$ when controlling the FWER with the Bonferroni correction as a multiple testing correction. When controlling the FDR with the Benjamini–Hochberg procedure, we report the hypotheses in the set $R_{H}^{BH}\cap R_{\tilde{H}}^{BH}$.

## 3. Results

This section presents the dataset under investigation to which we apply the proposed methodology (Section 3.1). An example of the observed *p*-value distributions for both $H_{j}$ and ${\tilde{H}}_{j}$ is presented in Section 3.2. We conclude with an example of the actual significances observed with our methodology (Section 3.3).

### 3.1. Dataset under Investigation

Severe lower respiratory tract infections (LRTI) are a leading cause of hospitalization and preventable death in children worldwide [16]. From 2010 to 2022, accounting for the United States alone, an average of 22.2 out of 100,000 children less than 18 years old were hospitalized with severe influenza virus infection, resulting in 1358 deaths [17]. Multiple organ dysfunction syndrome (MODS) is an uncommon but life-threatening complication of severe influenza infection in children that may negatively impact their longer-term health [1].

We consider a dataset originally created by the Pediatric Intensive Care Influenza (PICFLU) investigators group [2,3], comprising children of less than 18 years of age with confirmed influenza infection which were admitted to Pediatric Intensive Care Units (PICU) at 30 sites between March 2010 and March 2017. Influenza was confirmed as previously described in [18]. Individuals with known immunodeficiencies, chronic lung disease, symptomatic cardiac disease, neuromuscular disease, malignancy, metabolic or mitochondrial disease, or individuals who received systemic immunosuppressive medications within six weeks prior to admission for this acute illness were excluded.

A custom gene panel was designed for m=469 mRNA targets incorporating genes known for moderating inflammation, cytokines, and associated with influenza and sepsis. Based on similar studies, the dataset also includes seven housekeeping genes [19,20,21]. The gene panel of the PICFLU dataset is given in the Supplementary Material.

The Pediatric Sequential Organ Failure Assessment (pSOFA) score, ranging from 0 to 24, was used to identify MODS. It can also quantify MODS over time [22] and it is positively correlated with mortality. To be precise, organ dysfunction was defined by a pSOFA score of 2 or greater. The class “Prolonged MODS” was defined as multiple organ dysfunction and/or extracorporeal membrane support (ECMO), or death on or after PICU day 7. MODS was measured at the time of the initial sample collection. PICU survivors with MODS when the first sample was collected who did not have MODS on or after PICU day 7 were categorized as “MODS Recovery”.

The dataset under investigation contains n=45 individuals, divided into two groups *A* and *B* with $n_{1}=22$ and $n_{2}=23$ individuals, respectively.

Since the aim of this contribution is to showcase the new methodology, a simulated dataset is generated from the above dataset of the Pediatric Intensive Care Influenza (PICFLU) investigators group. As the analysis of the real dataset requires an extensive discussion of the biological implications, it is deferred to a separate publication. Instead, the simulated dataset we use was generated by bootstrapping (sampling with replacement) from the real data described above. For this, when bootstrapping, we keep the original group sizes of $n_{1}=22$ and $n_{2}=23$ individuals for groups *A* and *B*, respectively. For each individual, we consider the fixed panel of m=469 genes, for which we possess gene expression measurements at two endpoints.

The bootstrapping is conducted as follows. Since we are only interested in the contrasts, see Figure 1, we first compute the contrasts for both the group “Prolonged MODS” (group *A*) and “MODS Recovery” (group *B*) on the real data. While keeping fixed the original group sizes of $n_{1}=22$ and $n_{2}=23$ individuals for groups *A* and *B*, respectively, we pool all contrast measurements for the group “Prolonged MODS” (group *A*) and draw bootstrapped measurements from this pool to create a new panel of $n_{1}$ individuals and *m* genes. The same is carried out using the contrast data for the group “MODS Recovery” (group *B*). A vector of *m* new age measurements is created by sampling with replacement from the original *m* age measurements, which are all contained in the interval (0,16).

### 3.2. Example of p-Value Distributions for the Two Hypotheses

**Figure 3:**
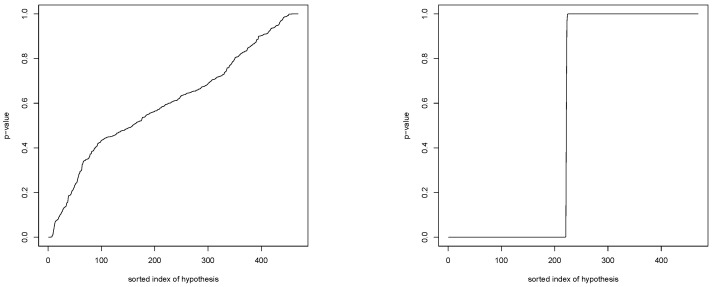
Sorted *p*-value distributions for $H_{j}$ (**left**) and ${\tilde{H}}_{j}$ (**right**).

Figure 3 shows the two distributions obtained after calculating the *p*-values for both $H_{j}$ and ${\tilde{H}}_{j}$ according to Section 2.4. Note that the *p*-values in both subfigures are sorted, meaning that it is not possible to immediately compare the *p*-values in the left and right subfigures. We observe that, for the test under the null, the *p*-values are quite conservative, with only very few significances. For the test under the alternative, we observe a step function behavior, in the sense that the level $\lambda_{j}$ we determine for each test ${\tilde{H}}_{j}$ with j∈{1,…,m} seems to make the *p*-values either (almost) zero or one.

### 3.3. Reported Genes

We evaluate all *p*-values considered in Section 3.2 by applying the Benjamini-Hochberg procedure to the ones calculated for the nulls $H_{j}$ and the alternatives ${\tilde{H}}_{j}$ separately at a level α/2, where α=0.05. As described in Section 3.3, we then look for any indices j∈{1,…,m} such that both $H_{j}$ and ${\tilde{H}}_{j}$ are rejected. For the above dataset, this procedure results in two genes to be reported which can be found in Table 1.

However, since the dataset we analyze here is created by bootstrapping from the PICFLU dataset (see Section 3.1), the *discovered* genes in Table 1 are actually random and thus not biologically meaningful. An application to real MODS data of [2,3], and the subsequent biological interpretation of the results, is deferred to a separate publication.

## 4. Discussion

This article considered the problem of testing contrasts for a gene expression application in the context of multiple organ dysfunction syndrome (MODS). The statistical challenge of the problem under consideration consists in the fact that we are interested in genes showing significances with respect to one group but *not* another group (denoted groups *A* and *B*). Although formulated as a problem with two endpoints, as a preprocessing step, the input data consisting of gene expression data collected at two different time points for the same set of genes and individuals in two different groups are converted to gene contrasts (differences in gene expression) per group. This effectively reduces the multiple endpoint problem to a single input.

Our proposed solution uses two hypothesis tests per gene under consideration, where *m* denotes the number of genes. Precisely, we conduct two linear regressions, where each linear regression allows us to determine if the contrasts can be explained by a single covariate alone (in our application this is the age covariate) and focus on the intercept to detect a departure from the baseline in gene expression. The two sets of *m* hypotheses (leading to a total of 2m hypotheses which are being tested) consist of *m* hypotheses under the null (to detect a departure from the baseline in group *A*) and *m* hypotheses under the alternative (to model the condition that we are interested in genes showing significances in group *A* but not in group *B*). Special attention is paid to the formulation and calibration of an appropriate level under the alternative. The level we choose is essentially arbitrary, but as motived in the literature (see Section 2.3), one option is to scale the intercept until it only explains a fraction (e.g., a fraction of 0.35) of the initial $R^{2}$ of the linear regression fit. To obtain *p*-values for both the null hypotheses and the alternative hypotheses, we give explicit formulas based on a t-distribution. We evaluate all *p*-values with the help of the FWER or FDR criterion to correct for multiple comparisons and report those genes as findings which are significant under both the null as well as the alternative.

There is no restriction on the number of gene contrasts that can be tested with our proposed methodology as long as the multiple testing correction is carried out correctly. This is due to the fact that the discovery of genes is essentially deferred to the discovery of significant hypotheses among the nulls $H_{j}$ and the alternative hypotheses ${\tilde{H}}_{j}$, see Section 2.5. As long as a valid testing procedure is being used, such as the one in [14] to control the FWER or the one in [15] to control the FDR, any number of gene contrasts can be tested. In particular, if a type I error of α is desired, it is valid to split this error evenly among the *m* nulls and the *m* hypotheses under the alternative, thus applying the Bonferroni correction at threshold α/2 to the *m* hypotheses per group.

The application which prompted the development of this methodology is in the area of multiple organ dysfunction syndrome (MODS). The dataset originally created for MODS stems from the Pediatric Intensive Care Influenza (PICFLU) investigators group [2,3] and serves as the basis of the experiments reported in this publication. However, the developed methodology is not tied to a certain type of data and, thus, we aimed to separate the presentation of the methodology and the analysis of the PICFLU dataset. Therefore, we used a simulated dataset to showcase the methodology, which is based on the real PICFLU dataset with an equal number of individuals and mRNA targets (as in the original PICFLU dataset) created via bootstrapping. Therefore, the *discovered* genes reported in Section 3 are actually not biologically meaningful and an interpretation of the *discoveries* is not sensible. This is due to the fact that an application to real MODS data will involve a much more meticulous biological interpretation of the results, and it is therefore deferred to a separate publication.

## Figures and Tables

**Table 1:** *p*-values for $H_{j}$ and ${\tilde{H}}_{j}$ observed for the two rejections with respect to the FDR criterion.

Name	*p*-Value for $H_{1}$	*p*-Value for $H_{2}$
ALAS1	6.240291 × 10${}^{-5}$	1.553433 × 10${}^{-51}$
CASP6	1.287339 × 10${}^{-4}$	1.127595 × 10${}^{-11}$

## Data Availability

All data analysed in this study are included in published articles of the Pediatric Intensive Care Influenza (PICFLU) investigators group [2,3].

## Supplementary Materials

The following supporting information can be downloaded at: https://www.mdpi.com/article/10.3390/genes14061134/s1, The gene panel of the PICFLU dataset is given in the supplementary material.
